# Enhancing pediatric pneumonia diagnosis through masked autoencoders

**DOI:** 10.1038/s41598-024-56819-3

**Published:** 2024-03-14

**Authors:** Taeyoung Yoon, Daesung Kang

**Affiliations:** Department of Healthcare Information Technology, Inje University, 197, Inje-ro, Gimhae-si, 50834 Korea

**Keywords:** Radiography, Respiratory distress syndrome

## Abstract

Pneumonia, an inflammatory lung condition primarily triggered by bacteria, viruses, or fungi, presents distinctive challenges in pediatric cases due to the unique characteristics of the respiratory system and the potential for rapid deterioration. Timely diagnosis is crucial, particularly in children under 5, who have immature immune systems, making them more susceptible to pneumonia. While chest X-rays are indispensable for diagnosis, challenges arise from subtle radiographic findings, varied clinical presentations, and the subjectivity of interpretations, especially in pediatric cases. Deep learning, particularly transfer learning, has shown promise in improving pneumonia diagnosis by leveraging large labeled datasets. However, the scarcity of labeled data for pediatric chest X-rays presents a hurdle in effective model training. To address this challenge, we explore the potential of self-supervised learning, focusing on the Masked Autoencoder (MAE). By pretraining the MAE model on adult chest X-ray images and fine-tuning the pretrained model on a pediatric pneumonia chest X-ray dataset, we aim to overcome data scarcity issues and enhance diagnostic accuracy for pediatric pneumonia. The proposed approach demonstrated competitive performance an AUC of 0.996 and an accuracy of 95.89% in distinguishing between normal and pneumonia. Additionally, the approach exhibited high AUC values (normal: 0.997, bacterial pneumonia: 0.983, viral pneumonia: 0.956) and an accuracy of 93.86% in classifying normal, bacterial pneumonia, and viral pneumonia. This study also investigated the impact of different masking ratios during pretraining and explored the labeled data efficiency of the MAE model, presenting enhanced diagnostic capabilities for pediatric pneumonia.

## Introduction

Pneumonia is an inflammatory lung condition primarily caused by bacteria, viruses, or fungal infections. It is characterized by the accumulation of fluid and cellular infiltrates in the alveoli, which can lead to symptoms such as fever, cough, shortness of breath, and chest pain^1^. While pneumonia can affect individuals of all ages, it presents specific difficulties in pediatric cases. The distinctive characteristics of respiratory systems in pediatric patients, coupled with the potential for rapid deterioration, make pneumonia particularly challenging. Diagnosing pediatric pneumonia is crucial for several reasons. Children under the age of 5, with immature immune systems, are more susceptible to infections including pneumonia. Prompt diagnosis and intervention are essential to prevent severe complications. In addition, pneumonia in children can progress rapidly, leading to respiratory distress and hypoxia. Early diagnosis is critical to initiate timely treatment and prevent further deterioration^2^. According to UNICEF, the annual estimated number of child deaths due to pneumonia exceeds 700,000, averaging about 2000 deaths per day^3^. The high mortality rate of pediatric pneumonia is mainly attributed to delayed diagnosis and excessive diagnostic costs in developing countries. Clinical symptoms of pediatric pneumonia can be non-specific and may overlap with other respiratory conditions, making it challenging to rely solely on clinical assessment for an accurate diagnosis.

Various diagnostic methods are available for pneumonia, including clinical assessment, laboratory tests, and imaging studies. Among these modalities, imaging, particularly chest X-rays, plays a crucial role in confirming the diagnosis. Chest X-rays are widely employed due to their ability to visualize the lungs and identify pulmonary abnormalities, consolidations, pleural effusions, and other signs indicative of pneumonia. They serve as a fundamental tool in the assessment and management of pneumonia, providing a non-invasive and rapid means of evaluating lung abnormalities and distinguishing between viral and bacterial pneumonia^4^. Despite the widespread use of chest X-rays for pneumonia diagnosis, there exist challenges. First, radiographic findings can be subtle and diverse, leading to interpretational ambiguity. Second, the varied clinical presentations of pneumonia may not always align with radiological findings. Third, subjectivity in readings, influenced by the observer's experience, introduces variability. Forth, especially in the case of children, complexity is increased due to anatomical differences and dynamic lung development. Finally, when conducting X-ray imaging on children, minimizing radiation exposure by reducing the radiation dose compromises diagnostic performance and prolongs diagnosis time^5,6^. These challenges highlight the need for advanced technologies such as deep learning to enhance diagnostic accuracy.

Deep learning has significantly revolutionized pneumonia diagnosis, offering significant contributions to the field. With the ability to process extensive amounts of medical imaging data, deep learning models excel in recognizing intricate patterns and abnormalities in chest X-rays associated with pneumonia. Numerous studies have delved into the use of deep learning models for diagnosing pneumonia through chest X-rays. In a study by Nishio et al. VGG16 was employed as a pretrained model and a combination of conventional techniques along with mixup as data augmentation methods were used to create and validate a computer-aided diagnosis (CAD) system^7^. This CAD system was specifically designed for classifying chest X-ray images into COVID-19 pneumonia, non-COVID-19 pneumonia, and healthy categories. The three-category accuracy of the CAD system reached 83.6%, encompassing COVID-19 pneumonia, non-COVID-19 pneumonia, and healthy classes, with a sensitivity exceeding 90% for COVID-19 pneumonia. Gupta et al. introduced a neural architecture search (NAS) approach to identify the optimal convolutional architecture for pneumonia detection in chest X-rays^8^. Their framework, named learning by teaching and inspired by human learning strategies, enhances a model's learning outcomes by motivating it to instruct other models to achieve optimal performance. Their results demonstrated a notable area under the ROC curve (AUC) of 97.6% for detecting pneumonia. Singh et al. introduced a quaternion channel-spatial attention network (QCSA) that combines spatial and channel attention mechanisms with a Quaternion residual network for the classification of chest X-ray images in pneumonia detection^9^. This integration allows the network to focus on significant regions in chest X-ray images while capturing intricate spatial features, ultimately enhancing pneumonia detection accuracy. Their approach achieved impressive results, with an accuracy of 94.53% and an AUC of 0.89 in pneumonia diagnosis. Yao et al. presented a two-stage attention-based multimodal model for pneumonia diagnosis^10^. The model consisted of three components: an attention-based image feature extraction module, a blood testing feature extraction module, and a modal fusion module. They integrated a local–global hybrid attention module to capture both local and deep global features in pneumonia images. The F1-scores for normal, bacterial, and viral were reported as 0.9333, 0.6721, and 0.3871, respectively.

Various studies have been conducted on diagnosing pneumonia using deep learning, but diagnosing pediatric pneumonia is considered relatively more challenging than diagnosing adult pneumonia. A major obstacle is the limited availability of labeled data for pediatric chest X-ray images with pneumonia. Training deep learning models requires a substantial amount of labeled data, and this constraint is particularly evident when dealing with pediatric pneumonia chest X-ray data, which comprises only a few thousand cases. To address the scarcity of labeled medical data, transfer learning, specifically fine-tuning pretrained models on large labeled datasets such as ImageNet, has been investigated as a potential solution^11^. Transfer learning offers advantages in improving generalization and performance, especially when the source and target data domains share similarities. However, challenges arise when applying transfer learning from models pretrained on natural images to medical data, including discrepancies in pixel value distribution, resolution, channel number, output label quantity, and other factors between the source and the medical data domain. As a result, features obtained from the source domain may not be the most suitable for medical data^12,13^.

One powerful approach to address the mentioned issues is self-supervised learning. Self-supervised learning learns meaningful representations from the intrinsic information in unlabeled data by solving a pretext task. This is achieved by automatically generating artificial supervisory signals from unlabeled data and employing these signals to pretrain a network through a pretext task. The pretrained network can then be fine-tuned on labeled target data through downstream tasks such as image classification. Self-supervised learning methods have exhibited comparable or superior performance when compared to conventional supervised learning or transfer learning methods^14–19^. In the medical domain, Azizi et al. demonstrated that self-supervised learning outperformed supervised learning pretrained with the ImageNet dataset in classifying dermatology skin conditions and multi-label chest X-ray classifications^20^. Xiao et al. adopted the masked autoencoder (MAE), a self-supervised learning method, due to its notable scalability, computational efficiency, and impressive performance in various vision tasks. They delved into the pretraining and fine-tuning procedures of MAE for the medical domain, achieving comparable performances on three chest X-ray datasets^21^.

This study aims to improve the diagnostic performance of pediatric pneumonia through self-supervised learning. Among various self-supervised learning methods, we focused our investigation on MAE due to its computational complexity and performance. To achieve this goal, the MAE backbone model was pretrained on 303,349 publicly available adult chest X-ray images. Following pretraining, the pretrained model was fine-tuned on pediatric chest X-ray data to further enhance its diagnostic capabilities for pediatric pneumonia.

The main contributions of this study can be outlined as follows:

1. Employing MAE, we pretrain the model on adult chest X-rays and then fine-tune it on pediatric chest X-rays to minimize domain discrepancies.
2. Beyond focusing on pediatric pneumonia diagnosis, our study extends its scope to differentiate between bacterial and viral pneumonia.
3. The proposed method demonstrates superior performance compared to fine-tuning ResNet-34 and ViT-S models with random weights or ImageNet weights on pediatric chest X-ray data.
4. Through various experiments, we investigate the impact of the masking ratio and labeled data fractions on the performance of MAE.

## Results

In this study, we conducted various experiments to demonstrate the effectiveness of pretraining the MAE model on adult chest X-ray images for diagnosing pediatric pneumonia. The adult chest X-ray images used to pretrain the MAE model were obtained from the publicly available ChestX-ray14 and CheXpert datasets^22,23^. To demonstrate the effectiveness of utilizing MAE for pretraining, we considered three methods to pretrain the backbone model. The first method involved training ResNet-34 and vision transformer (ViT) without pretraining, directly on pediatric pneumonia data with random weights. The second method entailed fine-tuning ResNet-34 and ViT pretrained on the ImageNet dataset with pediatric pneumonia data. The third method employed the MAE architecture, pretraining it with the ImageNet dataset.

Table 1 presents details about the datasets employed in this study. The pretraining datasets, ChestX-ray14 and CheXpert, consist of a total of 303,349 images. The fine-tuning dataset is the pediatric pneumonia chest X-ray dataset, consisting of 5232 training images and 624 test images^24^. Both training and test images are categorized into normal and pneumonia data, with the pneumonia data further being subcategorized into bacterial and viral types.

**Table 1:** Details on datasets utilized in this study: pretraining with ChestX-ray14 and CheXpert, fine-tuning with Pediatric Pneumonia Chest X-ray.

	Data name	Data category	Training data	Test data
Pretraining data	ChestX-ray14	–	112,120	–
	CheXpert	–	191,229	–
Fine-tuning data	Pediatric pneumonia chest X-ray	Normal	1349	234
		Bacterial	2538	242
		Viral	1345	148

**Figure 1:**
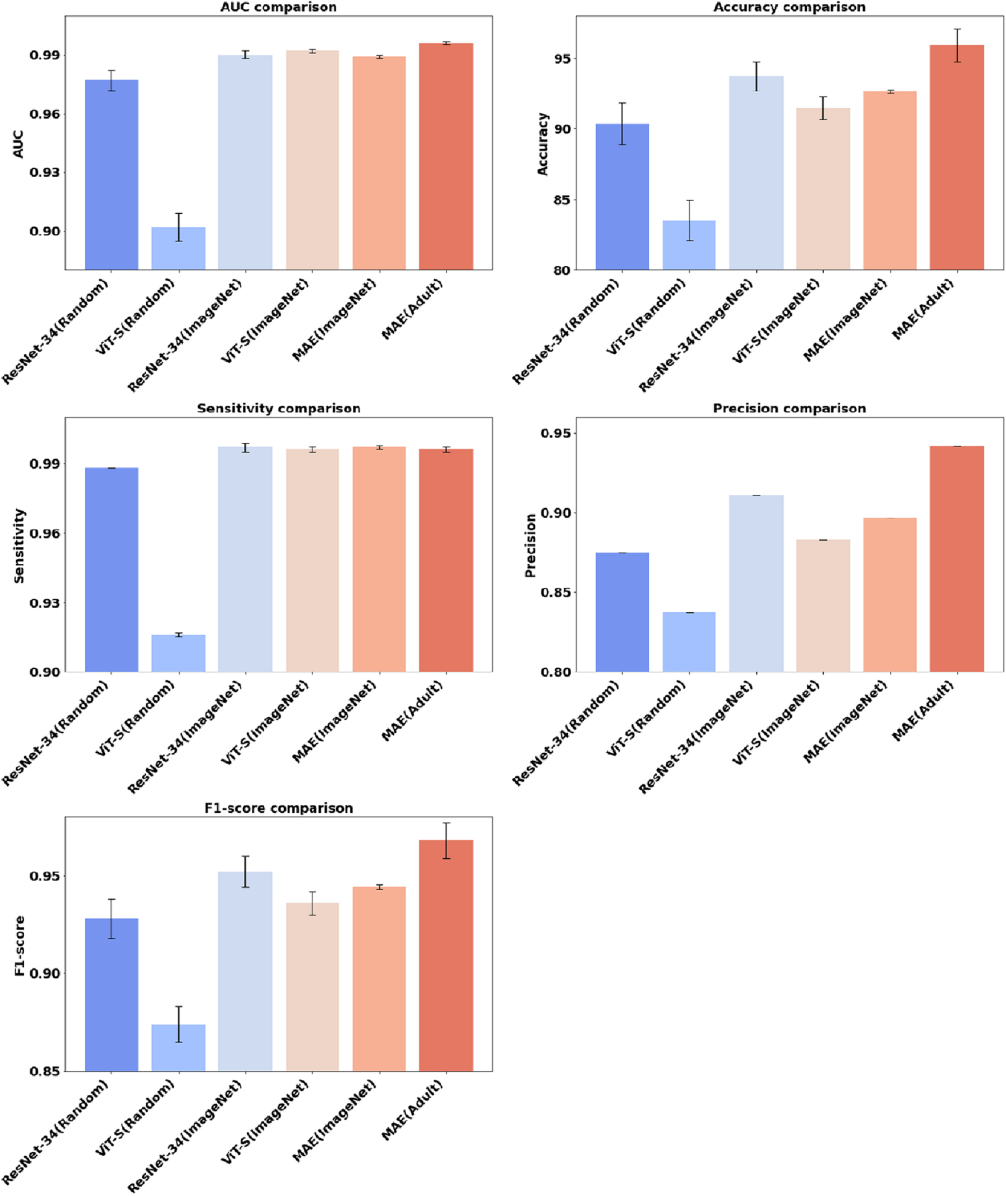
Performance comparison of MAE (Adult) model at masking ratio 0.95.

Table 2 and Fig. 1 present a comprehensive comparison of different pretraining methods and backbone models applied to pediatric pneumonia diagnosis. The chosen backbone models, ResNet-34 and ViT-S, were selected for their comparable parameter count, each totaling approximately 22 million. Models trained from scratch on pediatric data exhibited notable differences. ResNet-34 (Random) model outperformed the ViT-S (Random) model, achieving a higher AUC of 0.977 and accuracy of 90.38%, compared to ViT-S (Random) model's AUC of 0.902 and accuracy of 83.55%. This discrepancy may be attributed to the limited training data relative to ViT-S's model size and its lack of inductive bias. Leveraging pretrained weights from the ImageNet dataset improved the diagnostic capabilities of both ResNet-34 (ImageNet) and ViT-S (ImageNet). ResNet-34 (ImageNet) achieved an AUC of 0.990 and an accuracy of 93.70%, while ViT-S (ImageNet) obtained an AUC of 0.992 and an accuracy of 91.45%. The MAE (ImageNet) model, representing the MAE architecture pretrained on the ImageNet dataset, demonstrated competitive diagnostic metrics with an AUC of 0.989 and an accuracy of 92.63%. Interestingly, all three models pretrained on ImageNet exhibited similar performance metrics. The MAE (Adult) model, pretrained with adult chest X-ray images integrated with ChestX-ray14 and CheXpert datasets, demonstrated outstanding results. Despite the significantly smaller number of adult chest X-ray images compared to the ImageNet dataset, MAE (Adult) exhibited exceptional performance, with an AUC of 0.996 and an accuracy of 95.89%. Furthermore, the model demonstrated high sensitivity (0.996), precision (0.942), and F1-score (0.968), indicating its effectiveness in pediatric pneumonia diagnosis. The p values in Table 2 indicate the overall difference between MAE (Adult) and other methods. When comparing MAE (Adult) to other methods in terms of AUC, accuracy, precision, and F1-score, all have p values less than 0.05, indicating statistical significance. However, for sensitivity, comparing MAE (Adult) to ResNet (Random) and ViT (Random) yielded p values of 0.0290 and < 0.0001, respectively, indicating significant differences. On the other hand, when comparing MAE (Adult) to ResNet-34 (ImageNet), ViT-S (ImageNet), and MAE (ImageNet), the p values were 0.7658, 0.9871, and 0.5685, respectively, suggesting no significant differences.

**Table 2:** Performance metrics for classifying normal and pneumonia using various pretraining data and backbone models.

Pretraining data	Backbone models	AUC $($std)	Accuracy (std)	Sensitivity (std)	Precision (std)	F1-score (std)
Pediatric data (from scratch)	ResNet-34 (Random)	0.977 (0.005) p < 0.0001	90.38% (1.46) p < 0.0001	0.988 (0.000) p = 0.0290	0.875 (0.000) p < 0.0001	0.928 (0.010) p < 0.0001
	ViT-S (Random)	0.902 (0.007) p < 0.0001	83.55% (1.40) p < 0.0001	0.916 (0.001) p < 0.0001	0.837 (0.000) p < 0.0001	0.874 (0.009) p < 0.0001
ImageNet data	ResNet-34 (ImageNet)	0.990 (0.002) p = 0.0259	93.70% (1.05) p < 0.0001	**0.997 (0.002)** **p = 0.7658**	0.911 (0.000) p < 0.0001	0.952 (0.008) p < 0.0001
	ViT-S (ImageNet)	0.992 (0.001) p = 0.0007	91.45% (0.80) p < 0.0001	0.996 (0.001) p = 0.9871	0.883 (0.000) p < 0.0001	0.936 (0.006) p < 0.0001
	MAE (ImageNet)	0.989 (0.001) p = 0.0004	92.63% (0.13) p < 0.0001	**0.997 (0.001)** **p = 0.5685**	0.897 (0.000) p < 0.0001	0.944 (0.001) p < 0.0001
ChestX-ray14 + CheXpert data	MAE (adult)	**0.996 (0.001)**	**95.89% (1.17)**	0.996 (0.001)	**0.942 (0.000)**	**0.968 (0.009)**

p denotes the p values for comparing MAE (Adult). Highest performance values are in bold.

Table 3 presents the performance metrics of the MAE (Adult) model under different masking ratios. The masking ratio denotes the proportion of masked patches during pretraining. As the masking ratio increased from 0.65 to 0.95, the AUC consistently remains high at 0.996, indicating robust diagnostic capabilities. Accuracy also maintained a high level, ranging from 94.07 to 95.89%, demonstrating the model's ability to correctly classify pediatric pneumonia cases. Sensitivity, precision, and F1-score metrics exhibited similar trends across different masking ratios, with slight variations within acceptable ranges. Notably, at a masking ratio of 0.95, the model demonstrated its best performance. This indicates that the MAE (Adult) model performed effectively across various masking ratios, emphasizing its robustness and reliability in pediatric pneumonia diagnosis.

**Table 3:** Performance comparison at different masking ratio of MAE (Adult) model.

Masking ratio	AUC (std)	Accuracy (std)	Sensitivity (std)	Precision (std)	F1-score (std)
0.65	**0.996 (0.000)**	94.76% (0.67)	**0.997 (0.001)**	0.926 (0.000)	0.960 (0.005)
0.70	0.994 (0.001)	94.07% (0.86)	0.992 (0.002)	0.919 (0.000)	0.954 (0.006)
0.75	**0.996 (0.000)**	94.44% (1.05)	**0.997 (0.000)**	0.921 (0.000)	0.957 (0.008)
0.80	**0.996 (0.001)**	95.83% (0.57)	**0.997 (0.000)**	0.940 (0.000)	**0.968 (0.004)**
0.85	**0.996 (0.000)**	95.14% (0.20)	**0.997** (0.00**0)**	0.930 (0.000)	0.962 (0.001)
0.90	**0.996 (0.000)**	95.03% (0.39)	**0.997 (0.001)**	0.929 (0.000)	0.962 (0.003)
0.95	**0.996 (0.001)**	**95.89% (1.17)**	0.996 (0.001)	**0.942 (0.000)**	**0.968 (0.009)**

Highest performance values are in bold.

**Figure 2:**
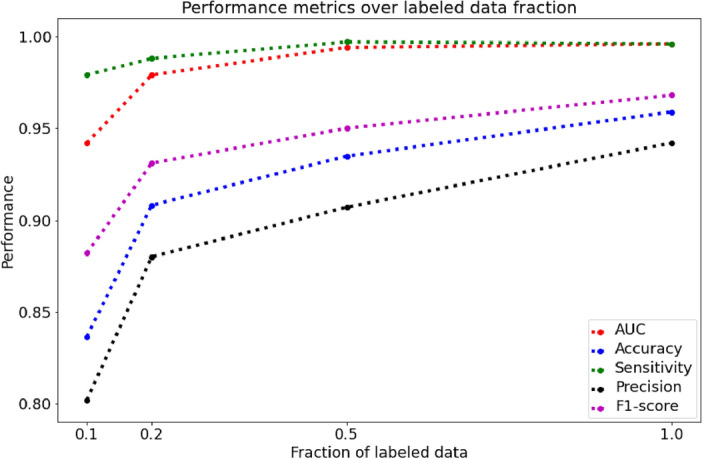
Performance comparison of MAE (Adult) model at masking ratio 0.95 across different fractions of labeled pediatric data. (The accuracy is represented as a value between 0 and 1, not as a percentage.).

Figure 2 illustrates the performance metrics of the MAE (Adult) model at a masking ratio of 0.95, corresponding to different fractions of labeled pediatric data. The fraction of labeled data indicates the proportion of pediatric data labeled during the fine-tuning process. As the fraction of labeled data increased from 10 to 100%, the model's performance metrics exhibited notable improvements. The AUC steadily rose from 0.942 to 0.996 showing enhanced diagnostic capabilities. Accuracy followed a similar upward trend, ranging from 0.836 to 0.959 (note: the accuracy values in Fig. 2 are represented as values between 0 and 1, not as percentages). Sensitivity, precision, and F1-score metrics also consistently improved with increasing fractions of labeled data. Overall, the results emphasized the positive impact of increasing labeled pediatric data on the model's performance, underscoring the importance of data quantity in fine-tuning process.

Table 4 provides a comprehensive overview of the performance metrics for classifying pediatric pneumonia into normal, bacterial pneumonia, and viral pneumonia categories using various pretraining data and backbone models. ResNet-34 (Random) achieved notable AUC values for normal (0.984), bacterial pneumonia (0.973), and viral pneumonia (0.923), with an accuracy of 90.88%. ViT-S (Random) showed inferior AUC values (84.47%) compared to ResNet-34 (Random), suggesting potential limitations in capturing subtle pediatric pneumonia patterns. ResNet-34 (ImageNet) demonstrated competitive performance with AUC values across all categories (normal: 0.994, bacterial pneumonia: 0.982, viral pneumonia: 0.942) and an accuracy of 92.61%. ViT-S (ImageNet) and MAE (ImageNet) exhibited similar performance, highlighting the advantages of pretraining on ImageNet. MAE (Adult) demonstrated exceptional performance with a masking ratio of 0.65, exhibiting high AUC values (normal: 0.997, bacterial pneumonia: 0.983, viral pneumonia: 0.956) and achieving an accuracy of 93.86%. Similarly, at a masking ratio of 0.95, MAE (Adult) maintained high AUC values (normal: 0.997, bacterial pneumonia: 0.983, viral pneumonia: 0.952) and attained an accuracy of 93.55%. These results highlight the model's effective capture of pediatric pneumonia patterns, emphasizing its robust diagnostic capabilities in pediatric pneumonia diagnosis across different masking ratios.

**Table 4:** Performance metrics for classifying pediatric chest X-ray data into normal, bacterial pneumonia, and viral pneumonia categories using different pretraining data and backbone models.

Pretraining data	Backbone models	Normal AUC (std)	Bacterial AUC (std)	Viral AUC (std)	Accuracy (std)
Pediatric data (from scratch)	ResNet-34 (Random)	0.984 (0.001)	0.973 (0.001)	0.923 (0.009)	90.88% (0.63)
	ViT-S (Random)	0.910 (0.003)	0.895 (0.003)	0.860 (0.003)	84.47% (0.24)
ImageNet data	ResNet-34 (ImageNet)	0.994 (0.000)	0.982 (0.001)	0.942 (0.001)	92.61% (0.70)
	ViT-S (ImageNet)	0.993 (0.001)	0.979 (0.003)	0.944 (0.001)	92.02% (0.45)
	MAE (ImageNet)	0.989 (0.001)	0.982 (0.003)	0.943 (0.005)	92.54% (0.22)
ChestX-ray14 + CheXpert data	MAE (adult) masking ratio 0.65	**0.997 (0.001)**	**0.983 (0.001)**	**0.956 (0.005)**	**93.86% (0.23)**
	MAE (Adult) masking ratio 0.95	**0.997 (0.001)**	**0.983 (0.001)**	0.952 (0.007)	93.55% (0.66)

Highest performance values are in bold.

## Discussion

In this study, we performed several experiments to evaluate the effectiveness of MAE in diagnosing pediatric pneumonia. This was accomplished by pretraining on adult chest X-ray datasets and subsequently fine-tuning on pediatric pneumonia chest X-ray datasets. The experiments yielded valuable insights into the application of MAE in medical imaging, highlighting the following key findings.

First and foremost, pretraining with adult chest X-ray images using MAE led to a significant improvement in pediatric pneumonia diagnosis compared to pretraining with ImageNet or without any pretraining. These results can be attributed to considerations from both architectural and data perspectives. Architecturally, the MAE encoder is designed to extract global representations from partial observations, while the decoder learns local representations by reconstructing missing pixels. This unique architecture allows MAE to effectively capture both local and global features of chest X-ray images, resulting in outstanding performance after fine-tuning. From a data viewpoint, a crucial factor is the minimal domain discrepancy between the adult chest X-ray data used for pretraining and the pediatric chest X-ray data used for fine-tuning. Recent research suggests that out-of-domain pretraining may not be beneficial for network initialization, possibly due to domain shift. Optimal performance can be achieved through pretraining and fine-tuning under in-domain transfer^21,25^. Despite not being exact match in-domain data between adult chest X-ray and pediatric chest X-ray images, the significant similarities in structural and textural features shared between them seem to contribute significantly to the impressive performance observed in this study.

Secondly, the performance of the MAE model on chest X-ray images was influenced by the masking ratio applied during pretraining. Determining the optimal masking ratio involves considerations of information redundancy in the data. Notably, while BERT employed a 15% masking ratio for language tasks, MAE utilized a 75% masking ratio for image-related tasks^17,26^. Due to the substantial similarity in chest anatomy, chest X-rays inherently exhibit higher information redundancy compared to natural images. Consequently, MAE has favored a 90% masking ratio specifically for chest X-rays^21^. In our experiments, we systematically varied the masking ratios from 65 to 95%, incrementing by intervals of 5%. As detailed in Tables 3 and 4, the optimal masking ratio for classifying normal and pediatric pneumonia was found to be 95%, while for classifying normal, bacterial pneumonia, and viral pneumonia, the optimal masking ratio was 65%. Despite the task-specific variations in optimal masking ratios, higher masking ratios allowed the efficient learning of the ViT encoder with a small number of masked patches. This contributed to reduced time and memory complexities in the learning process.

Lastly, this study demonstrates that MAE operates as a labeled data-efficient model, maintaining comparable performance even when trained on a partial dataset. To experimentally illustrate this, we randomly partitioned the pediatric chest X-ray dataset into subsets of 10%, 20%, 50%, and 100%. As depicted in Fig. 2, the model's performance exhibited improvement with an increasing percentage of labeled pediatric data. These findings suggest that pretraining with adult chest X-ray images using MAE proves to be beneficial for fine-tuning models with limited pediatric chest X-ray images. Typically, training large deep learning models with limited data presents challenges and risks of overfitting due to the abundance of parameters in the model. However, pretraining with MAE enables the model to establish a robust representation of the target dataset, effectively mitigating these challenges. This finding emphasizes the potential applicability of MAE in settings where obtaining large quantities of labeled data is challenging, such as clinical environments. Furthermore, employing a smaller training dataset for training a large deep learning model reduces labeling costs, a significant benefit considering the conventional requirement for a large amount of labeled data in traditional supervised deep learning training.

In this study, the pediatric pneumonia chest X-ray data used for fine-tuning consists of an imbalanced distribution, with 1349 images in the normal category and 3883 images in the pneumonia category. The results discussed earlier were obtained without considering data balancing issue. The pneumonia classification results considering the imbalance data issue are provided in Table S1 of the supplementary materials. To address this, a weighted loss function was implemented by modifying the standard loss function during model fine-tuning phase. In this approach, higher weights were assigned to the minority class (normal category) and lower weights to the majority class (pneumonia category). Specifically, the weights were adjusted to be inversely proportional to the frequency of the classes, ensuring that the minority class received a higher weight while the majority class received a lower weight. When comparing Table S1 and Table 2, both tables show similar performance values and performance trends across various pretraining datasets and backbone models. These results demonstrate comparable levels of AUC, accuracy, sensitivity, precision, and F1-score and suggest consistency in observed patterns. Notably, the reported MAE (Adult) performance values in both tables are almost identical, with standard deviations falling within a similar range. This suggests that the models perform similarly across the two datasets.

To demonstrate the effectiveness of MAE (Adult), we compared its performance with prominent self-supervised learning methods, namely MOCO-v2 and BYOL^15,16^. The training strategy for both MOCO-v2 and BYOL was identical to that of MAE (Adult), involving pretraining with adult chest X-rays exhibiting similar shapes and textures to pediatric chest X-rays, along with minimal domain discrepancy, followed by fine-tuning with pediatric chest X-rays. The results obtained from MOCO and BYOL have been included in Table S2 of the supplementary materials. MOCO-v2 achieved AUC, accuracy, sensitivity, precision, and F1-score values of 0.973, 90.87%, 0.988, 0.881, and 0.931, respectively. When compared to MOCO-v2, MAE (Adult) generally demonstrates higher values across performance metrics. As for BYOL, its AUC, accuracy, sensitivity, precision, and F1-score values are 0.988, 96.05%, 0.998, 0.942, and 0.969, respectively. In comparison to BYOL, MAE (Adult) exhibits a higher AUC while their precision values are identical. Additionally, although BYOL demonstrates slightly better accuracy, sensitivity, and F1-score values than MAE (Adult), the differences are minimal. MOCO-v2, on the other hand, exhibits lower overall performance compared to BYOL and MAE (Adult), possibly due to weak augmentation. Similar to SimCLR, MOCO-v2 requires strong augmentation^14,15^. However, in this study, MOCO-v2 employs weak augmentation to mitigate potential risks, such as cropping or introducing bias to critical elements like informative lesions or organs in medical images. Therefore, it appears to perform less effectively compared to other self-supervised learning methods. Although our comparison is limited to some self-supervised learning approaches, we believe these results sufficiently demonstrate the utility of MAE (Adult).

Several studies have investigated pediatric pneumonia diagnosis using the same dataset and the same official split of pediatric pneumonia chest X-rays employed in this study. Table 5 outlines the research methodologies and performance disparities between this study and others. Kermany et al. fine-tuned the Inception-v3 model, pretrained on the ImageNet dataset, achieving an AUC of 0.968 and an accuracy of 92.80%^24^. Liang and Zheng designed a custom residual network, pretrained on the ChestX-ray14 dataset and fine-tuned with pediatric pneumonia data, resulting in an AUC of 0.953 and an accuracy of 90.50%^5^. Ayan et al. employed ensemble learning, selecting the top-performing CNN models (ResNet-50, MobileNet, and Xception) from the seven models pretrained on the ImageNet dataset. The ensemble achieved an AUC of 0.917 and an accuracy of 93.26%^27^. Mabrouk et al. utilized ensemble learning with DenseNet169, MobileNetV2, and ViT networks pretrained on ImageNet, reaching an accuracy of 93.91%^28^. Kiliçarslan et al. proposed a 4-layer CNN with a superior exponential activation function, achieving an accuracy of 95.37% with randomly initialized weights^29^. Gazda et al. conducted experiments similar to our study, employing contrastive learning to pretrain the ResNet-50 network with CheXpert data. The pretrained network was then fine-tuned with pediatric pneumonia data, resulting in an AUC of 0.977 and an accuracy of 91.50%^30^. Singh et al. fine-tuned the DEIT_B model pretrained on the ImageNet dataset. We replicated the results using the same official data split as ours, yielding an AUC of 0.995 and an accuracy of 94.50%^31^. Nisho et al. fine-tuned the EfficientNet with the noisy student network pretrained on the ImageNet dataset. However, they conducted experiments using the COVID-19 pneumonia dataset, which differs from our experiment. Reproducing the results with the pediatric pneumonia chest X-rays dataset using EfficientNet-B4 with the noisy student model, we achieved an AUC of 0.991 and an accuracy of 93.94%^32^. Our approach demonstrates superior performance with an AUC of 0.996 and an accuracy of 95.89%, highlighting its effectiveness compared to the referenced studies. This suggests promising potential for the proposed approach in the context of diagnosing pediatric pneumonia with pediatric chest X-ray images.

**Table 5:** Performance of other recent works on the pediatric pneumonia chest X-ray dataset.

Authors	Approaches	AUC	Accuracy (%)
Kermany et al.^24^	Inception-v3 network pretrained on ImageNet dataset	0.968	92.80
Liang & Zheng^5^	Customized residual network pretrained on ChestX-ray14 dataset	0.953	90.50
Ayan et al.^27^	Ensemble learning of ResNet-50, MobileNet, Xception networks pretrained on ImageNet dataset	0.917	93.26
Mabrouk et al.^28^	Ensemble learning of DenseNet169, MobileNetV2, and ViT networks pretrained on ImageNet dataset	-	93.91
Kiliçarslan et al.^29^	4-Layer CNN network with superior exponential activation function from scratch	-	95.37
Gazda et al.^30^	Pretraining is achieved through the contrastive learning approach with CheXpert data	0.977	91.50
Singh et al.^31^	Deit-B model pretrained on ImageNet dataset	0.995^†^	94.50^†^
Nishio et al.^32^^†^	EfficientNet-B4 with noisy student network pretrained on ImageNet dataset	0.991^†^	93.94^†^
Proposed	Pretraining the MAE model with integrated CheXpert and ChestX-ray14 dataset	0.996	95.89

^†^Reproduced results using the same dataset and the official data split of pediatric pneumonia chest X-rays.

## Conclusion

In this study, we proposed an approach to enhance the diagnosis of pediatric pneumonia using masked autoencoder. The methodology involved pretraining the ViT model with adult chest X-ray images, followed by fine-tuning with pediatric chest X-ray data. The experimental findings demonstrated that the MAE model, pretrained with adult chest X-ray images, achieved superior performance in diagnosing pediatric pneumonia, with an accuracy of 95.89%, an AUC of 0.996, a sensitivity of 0.996, a precision of 0.942, and an F1-score of 0.968. Moreover, the classification of pediatric chest X-ray data into normal, bacterial pneumonia, and viral pneumonia categories resulted in an accuracy of 93.86%, with individual AUC values of 0.997 for normal, 0.983 for bacterial, and 0.956 for viral. These outcomes, compared to other methods, emphasize the effectiveness of our proposed approach. This emphasizes the significance of our method in the medical imaging field, particularly in scenarios where labeled data is limited. The promising outcomes anticipate the broader applicability of our proposed method across diverse medical imaging domains in the future.

## Materials and methods

### Adult chest X-ray datasets and a pediatric chest X-ray pneumonia dataset

We employed two datasets, ChestX-ray14 and CheXpert, for the pretraining of MAE models. The ChestX-ray14 dataset comprises 112,120 frontal view chest X-ray images. Among these, 51,708 exhibit one or more pathologies across 14 pathology classes, while the remaining 60,412 images do not show any signs of disease^22^. The CheXpert dataset consists of 224,316 chest radiographs, including both frontal and lateral views. The dataset is annotated for 14 observations covering 12 pathologies, support devices, and observations with no findings. Specifically, we selectively utilized only frontal view images from the CheXpert dataset, totaling 191,229 images^23^. Despite both datasets having labels, but we chose not to use them. The final number of images used for MAE pretraining is the sum of both datasets, amounting to 303,349 images.

The pediatric chest X-ray data used in the study is from the Pediatric Pneumonia Chest X-ray dataset^24^. This dataset consists of 5856 pediatric chest X-ray images, which were divided into training and test datasets. Of the total, 5232 images (1349 normal, 3883 pneumonia) were officially assigned to the training set, while the remaining 624 images (234 normal, 390 pneumonia) formed the test set. Eighty percent of the training data was employed for either fine-tuning the pretrained networks or training networks from scratch, while the rest of the training data was used as validation data.

### Pretraining with adult chest X-ray images

**Figure 3:**
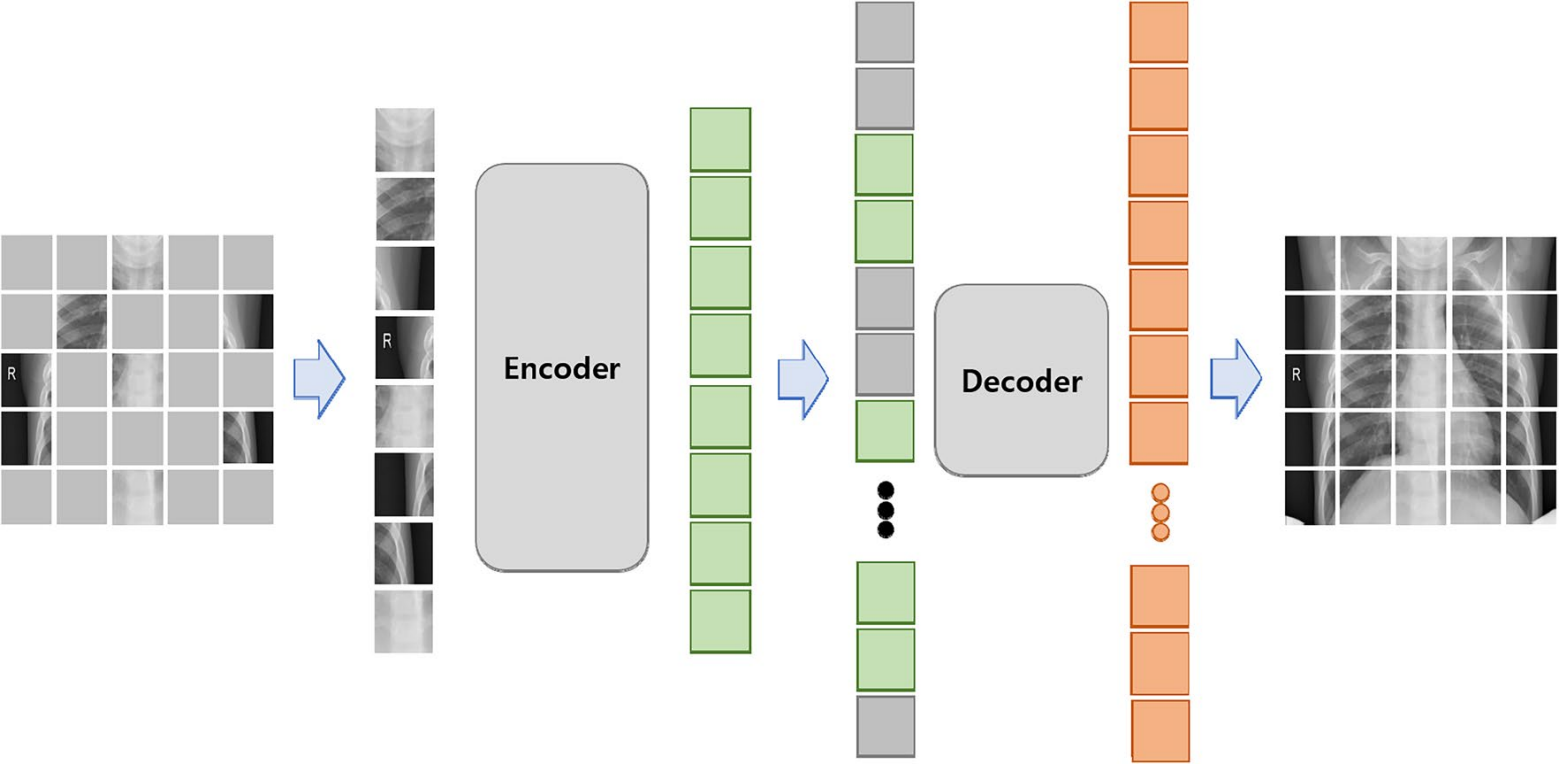
Architecture of MAE.

Recently, MAE has proven to be effective in pretraining vision transformers (ViT) for the images classification, detection and segmentation tasks^17,33,34^. MAE, a type of denoising autoencoder, is specifically designed to reconstruct the original signal from partial observations. Illustrated in Fig. 3, MAE features an asymmetric encoder-decoder architecture. The MAE encoder, a variant of ViT, exclusively processes visible, unmasked patches. By reconstructing entire images from partially masked inputs, it aggregates contextual information, allowing for the inference of masked image regions^17^. In medical imaging tasks, we assert that contextual information plays a crucial role in reconstructing masked image patches, given the inherent dependence and connection of region of interest (ROI) with its physiological environment and surroundings. In this study, we employed the ChestX-ray14 and CheXpert datasets as inputs for the MAE encoder. The adult chest X-ray images were resized to 256 × 256 pixels and standardized using mean and standard deviation calculated from ImageNet. Following this, we applied random resizing cropping (scale range 0.5–1.0) to 224 × 224 pixels and horizontal flipping for dataset augmentation. However, to prevent the potential risk of cropping or introducing bias to informative lesions or organs, crucial aspects in medical images, no additional data augmentation methods were applied. The preprocessed images were partitioned into non-overlapping 16 × 16 patches. Each input patch was assigned a token through linear projection, with an additional positional embedding. Subsequently, a randomly selected subset, ranging from 65 to 95% of these tokens, was masked. As the MAE encoder exclusively deals with visible and unmasked tokens (5–35%), we achieved efficient pretraining with reduced computational and memory demands. The MAE encoder is encouraged to extract a global representation from partial observations, as its output tokens are employed in the MAE decoder to reconstruct the learnable masked tokens. The MAE decoder processes a full set of tokens by combining encoded visible tokens with learnable mask tokens. Incorporating positional embeddings into all input tokens, the decoder reconstructs patches at their specific masked positions, reshaping the output to generate a reconstructed image. It's important to note that the MAE decoder is employed exclusively during the pretraining process and is intentionally designed to be smaller than the encoder to optimize pretraining efficiency. The MAE is trained using a reconstruction loss, specifically mean squared error between reconstructed and original images. Notably, the loss computation is confined to the masked patches. For optimizing the MAE network, we adopted the AdamW optimizer with parameters (${\beta }_{1}=0.9, {\beta }_{2}=0.95$) and a weight decay of 0.05. The transformer blocks in ViT were initialized using Xavier uniform initialization. The initial learning rate and batch size were set to 1.5e−4 and 128, respectively. The learning rate was warmed up for the initial 20 epochs and was adjusted using a cosine annealing schedule. The MAE pretraining process ran for 800 epochs. The pseudocode for MAE is provided in Table S3 of the supplementary materials.

### Fine-tuning with pediatric chest X-ray images

In contrast to natural images found in ImageNet dataset, there exists minimal domain discrepancy between adult and pediatric chest X-ray images. Specifically, adult chest X-ray images share significant similarities with pediatric chest X-ray images, encompassing both structural and textural aspects. We hypothesize that despite the number of adult chest X-ray images being 1/42 of the ImageNet training data, pretraining with adult chest X-ray images can yield better performance than pretraining with the ImageNet dataset due to the domain congruity between adult and pediatric chest X-ray images. In other words, domain congruity between upstream and downstream data plays a key role in effective weight initialization, ultimately influencing the network's performance during fine-tuning. To validate this hypothesis, we conducted fine-tuning on pediatric chest X-ray images using two different approaches: one utilizing weights pretrained on ImageNet dataset and the other using weights pretrained on adult chest X-ray images with MAE. Fine-tuning was carried out using an end-to-end approach on a labeled dataset consisting of 5232 pediatric chest X-ray images. Model optimization employed the AdamW optimizer (${\beta }_{1}=0.9, {\beta }_{2}=0.95$) with a weight decay of 0.05. The initial learning rate and batch size were set to 2.5e−3 and 128, respectively, with a cosine annealing schedule. Following the recommendations in^21^, we applied layer-wise LR decay of 0.55, RandAug magnitude of 6, and a DropPath rate of 0.2. The fine-tuning process ran for 75 epochs with a warm-up period of 5 epochs. For each set of experiments, we repeated the same procedure three times, using different random seeds for weight initialization. After fine-tuning, the fine-tuned model was used to evaluate 624 test images.

### Performance measures and statistical analysis

To assess the discriminative capability of the fine-tuned models, we computed overall classification accuracy (ACC), sensitivity (SEN), precision (PRE), F1-score, and the area under the receiver operating characteristic curve (AUC) for quantitative evaluation. The AUC represents the computed values of 1-specificity and sensitivity^35^. The mathematical formulas for the mentioned measures are provided below:

$$ACC=\frac{TP+TN}{TP+FP+FN+TN}\times 100\%$$

$$SEN=\frac{TP}{TP+FN}$$

$$PRE=\frac{TP}{TP+FP}$$

$$F1-score=2\times \left(\frac{SEN\times PRE}{SEN+PRE}\right)$$

$$AUC=area\, under\, the\, ROC\, curve$$

Here, TP, FP, FN, and TN denote the true positive, false positive, false negative, and true negative, respectively.

When comparing performance measures (AUC, accuracy, sensitivity, precision, F1-score) among the algorithms in Table 2, we computed bootstrap confidence intervals (CIs) to assess correlations among the values within the same pediatric subject. Each correlation was determined by computing a bootstrap CI, where pairs of observations were sampled with replacement independently in each group. p values were obtained using 1000 replications of bootstrapping.

### Supplementary Information

Supplementary Information.

## Data Availability

The data utilized in this study is openly accessible to the public. The ChestX-ray14 dataset is available at https://nihcc.app.box.com/v/ChestXray-NIHCC, and the CheXpert dataset can be obtained from https://stanfordmlgroup.github.io/competitions/chexpert/. The Pediatric Pneumonia Chest X-ray data can be downloaded from https://www.kaggle.com/datasets/andrewmvd/pediatric-pneumonia-chest-xray.
