# Supplementary material for: Enhancing pediatric pneumonia diagnosis through masked autoencoders
Source: Sci Rep. 2024 Mar 14;14:6150. doi: 10.1038/s41598-024-56819-3 (PMC10937919; doi:10.1038/s41598-024-56819-3)
Supplement: Supplementary file 1 — Supplementary Information. [file 41598_2024_56819_MOESM1_ESM.docx]

**Enhancing Pediatric Pneumonia Diagnosis Through Masked Autoencoder**

Taeyoung Yoon, Daesung Kang*****

Department of Healthcare Information Technology, Inje University, Gimhae-si, Republic of Korea

Table S1. Performance metrics with weighted loss function for classifying normal and pneumonia using various pretraining data and backbone models. (Highest performance values are in bold.)

| Pretraining data | Backbone models | AUC (std) | Accuracy (std) | Sensitivity (std) | Precision (std) | F1-score (std) |
| --- | --- | --- | --- | --- | --- | --- |
| Pediatric data (from scratch) | ResNet-34 (Random) | 0.983 (0.002) | 91.29% (0.46) | 0.995 (0.002) | 0.881 (0.000) | 0.935 (0.003) |
|  | ViT-S (Random) | 0.910 (0.002) | 84.99% (0.27) | 0.948 (0.015) | 0.835 (0.000) | 0.888 (0.001) |
| ImageNet data | ResNet-34 (ImageNet) | 0.988 (0.001) | 93.06% (1.83) | **1.000 (0.000)** | 0.901 (0.000) | 0.948 (0.013) |
|  | ViT-S (ImageNet) | 0.993 (0.001) | 92.47% (0.57) | 0.996 (0.001) | 0.896 (0.000) | 0.943 (0.004) |
|  | MAE (ImageNet) | 0.986 (0.003) | 91.45% (0.62) | 0.997 (0.001) | 0.882 (0.000) | 0.936 (0.004) |
| ChestX-ray14  + CheXpert data | MAE (Adult) | **0.997 (0.000)** | **95.19% (0.23)** | 0.997 (0.000) | **0.931 (0.000)** | **0.963 (0.002)** |

Table S2. Performance comparison of self-supervised learning methods on pediatric pneumonia data.

| Pretraining data | Self-supervised learning models | AUC (std) | Accuracy (std) | Sensitivity (std) | Precision (std) | F1-score (std) |
| --- | --- | --- | --- | --- | --- | --- |
| ChestX-ray14 + CheXpert data | MOCO-v2 | 0.973 (0.009) | 90.87% (2.18) | 0.988 (0.001) | 0.881 (0.000) | 0.931 (0.015) |
|  | BYOL | 0.988 (0.007) | **96.05% (0.15)** | **0.998 (0.002)** | **0.942 (0.000)** | **0.969 (0.001)** |
|  | MAE (Adult) | **0.996 (0.001)** | 95.89% (1.17) | 0.996 (0.001) | **0.942 (0.000)** | 0.968 (0.009) |

Table S3. The pseudocode of MAE

| Pseudocode of MAE in a PyTorch-like style. |
| --- |
| $X_{in}$ : input image  ${Patch}_{E}$ : image to patches  $proj$ : linear projection  ${Pos}_{E}$ : positional embeddings in Encoder  ${R_{M}}_{patch}$ : patch shuffle and random masking  $mask_{ratio}$ : masking ratio (65~95%)  $X_{um}$ : unmasked tokens  $X_{m}$ : masked tokens  $E_{r}$ : Encoder (based on ViT-S)  ${Pos}_{D}$ : positional embeddings in Decoder  $D_{r}$ : Decoder (based on ViT-S) |
| # Encoder  $X={Patch}_{E}\left( X_{in} \right)$ # make image into patches$X=proj(X)+{Pos}_{E}$ # patches to tokens via linear projection with positional embedding  $X_{um}, X_{m}={R_{M}}_{patch}\left( X, mask_{ratio} \right)$ # random masking based on masking ratio  $X_{um}=E_{r}\left( X_{um} \right)$ # input the unmasked tokens to the Encoder  # Decoder  $X=X_{m}+X_{um}$ # concatenate masked and unmasked tokens  $X=X+{Pos}_{D}$ # add positional embeddings to the full set of tokens  $X_{out}=D_{r}(X)$ # input the tokens to the Decoder  # Calculate Loss  $loss=\left( X_{out}- X_{in} \right)**2$ $loss=loss.mean\left( -1 \right)$ $loss=\left( loss\times X_{m} \right).sum()/X_{m}.sum()$ |
